# Supplementary material for: Current induced electromechanical strain in thin antipolar Ag2Se semiconductor
Source: Nat Commun. 2025 Feb 20;16:1818. doi: 10.1038/s41467-025-57057-5 (PMC11842724; doi:10.1038/s41467-025-57057-5)
Supplement: Supplementary file 1 — Supplementary Information [file 41467_2025_57057_MOESM1_ESM.pdf]

Supplementary Information for

Current induced electromechanical strain in thin antipolar Ag_2Se semiconductor

Hao Luo[1,3†], Qi Liang[1,3†], Anan Guo[1,3], Yimeng Yu[1,3], Haoyang Peng[1,3], Xiaoyi Gao[1], Yihao Hu[2], Xianli Su[1], Ctirad Uher[4], Yu Zheng[1], Dongwang Yang[1], Xiaolin Wang[5], Qingjie Zhang[1], Xinfeng Tang[1], Shi Liu[2], Gustaaf Van Tendeloo[3,6], Shujun Zhang[5*] and Jinsong Wu[1,3*]

[1]*State Key Laboratory of Advanced Technology for Materials Synthesis and Processing, Wuhan University of Technology, Wuhan 430070, China*

[2]*Key Laboratory for Quantum Materials of Zhejiang Province, Department of Physics, School of Science and Research Center for Industries of the Future, Westlake University, Hangzhou, Zhejiang 310030, China*

[3]*Nanostructure Research Center, Wuhan University of Technology, Wuhan 430070, China*

[4]*Department of Physics, University of Michigan, Ann Arbor, Michigan 48109, USA*

[5]*Institute for Superconducting and Electronic Materials, Faculty of Engineering and Information Sciences, University of Wollongong, Wollongong, NSW 2500, Australia*

[6]*EMAT (Electron Microscopy for Materials Science), University of Antwerp, Belgium*

This file includes:

Supplementary Figs. 1 to 23
Supplementary note 1
Supplementary References

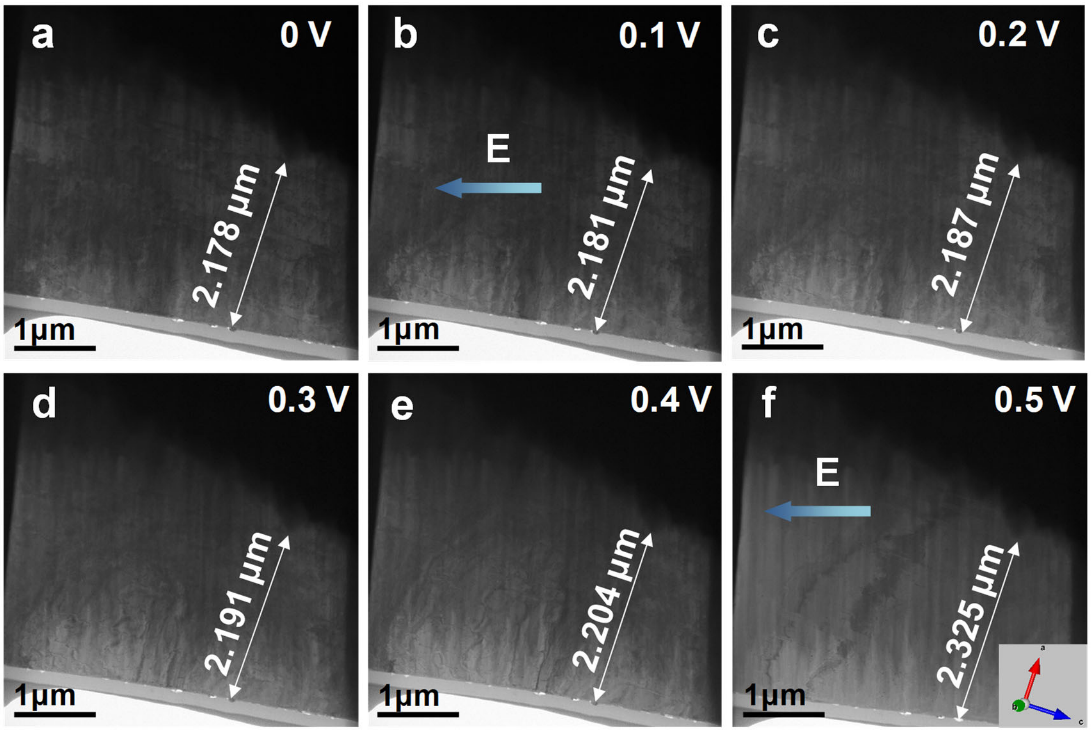

Figure S1. Microstructural evolution of Ag_2Se thin film under an increasing electric field, showing a mechanic stretch along the [001] direction (referring to α-Ag_2Se). The TEM images taken when the applied voltage is (a) 0V, (b) 0.1 V, (c) 0.2V, (d) 0.3V, (e) 0.4V and (f) 0.5V, respectively. It shows ~0.1% strain at 0.1V, ~0.4% strain at 0.2V, ~0.6% strain at 0.3V, ~1.2% strain at 0.4V, and ~6.7% strain at 0.5V.

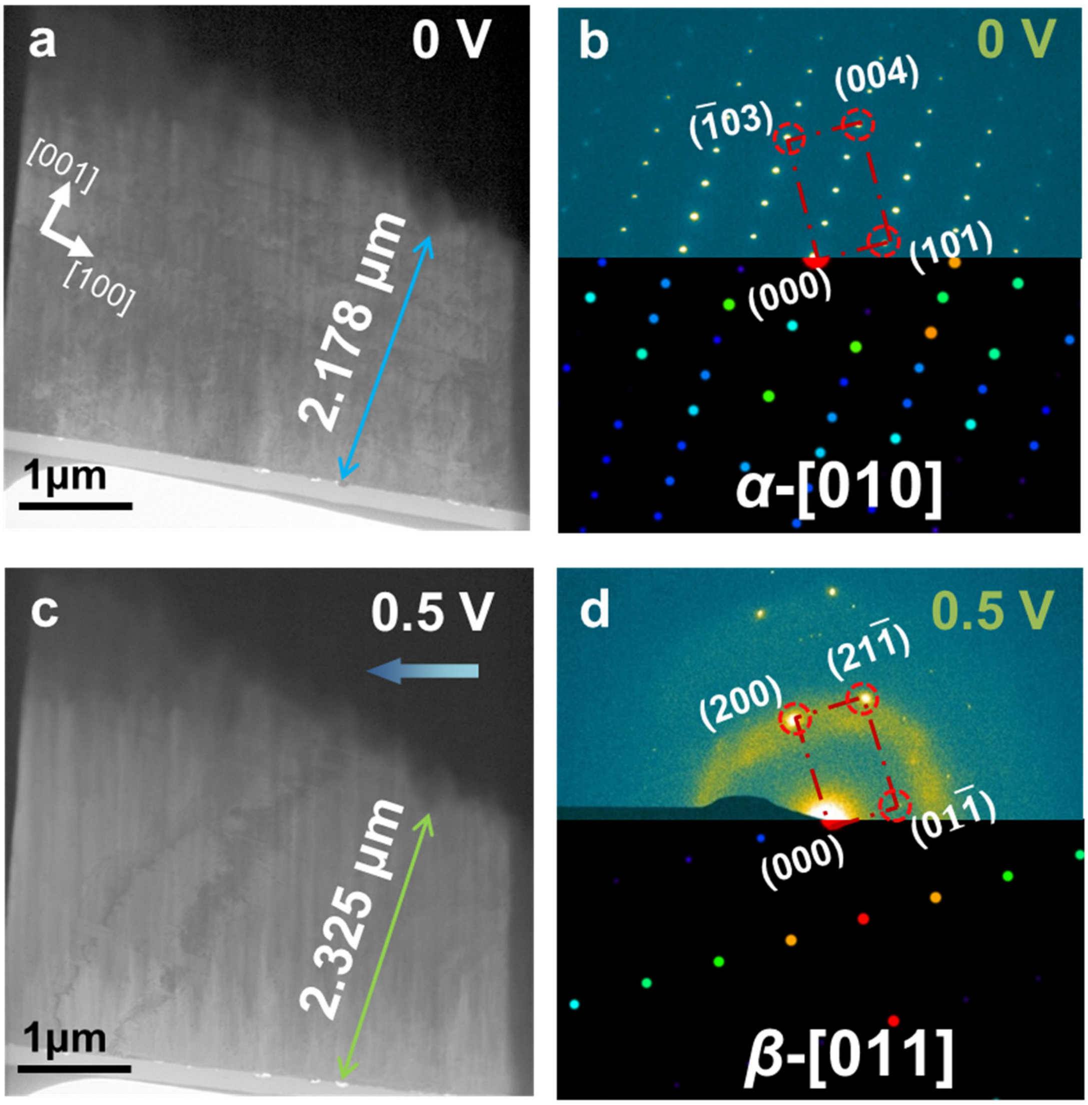

Figure S2. Phase transition of *α*-Ag_2Se to *β*-Ag_2Se at a relatively high electric field, accompanied by large a mechanic stretch along the [001] direction (referring to *α*-Ag_2Se). The TEM images are taken when the applied voltage is (a) 0V and (c) 0.5V, respectively. (d), (e) The corresponding SAED patterns.

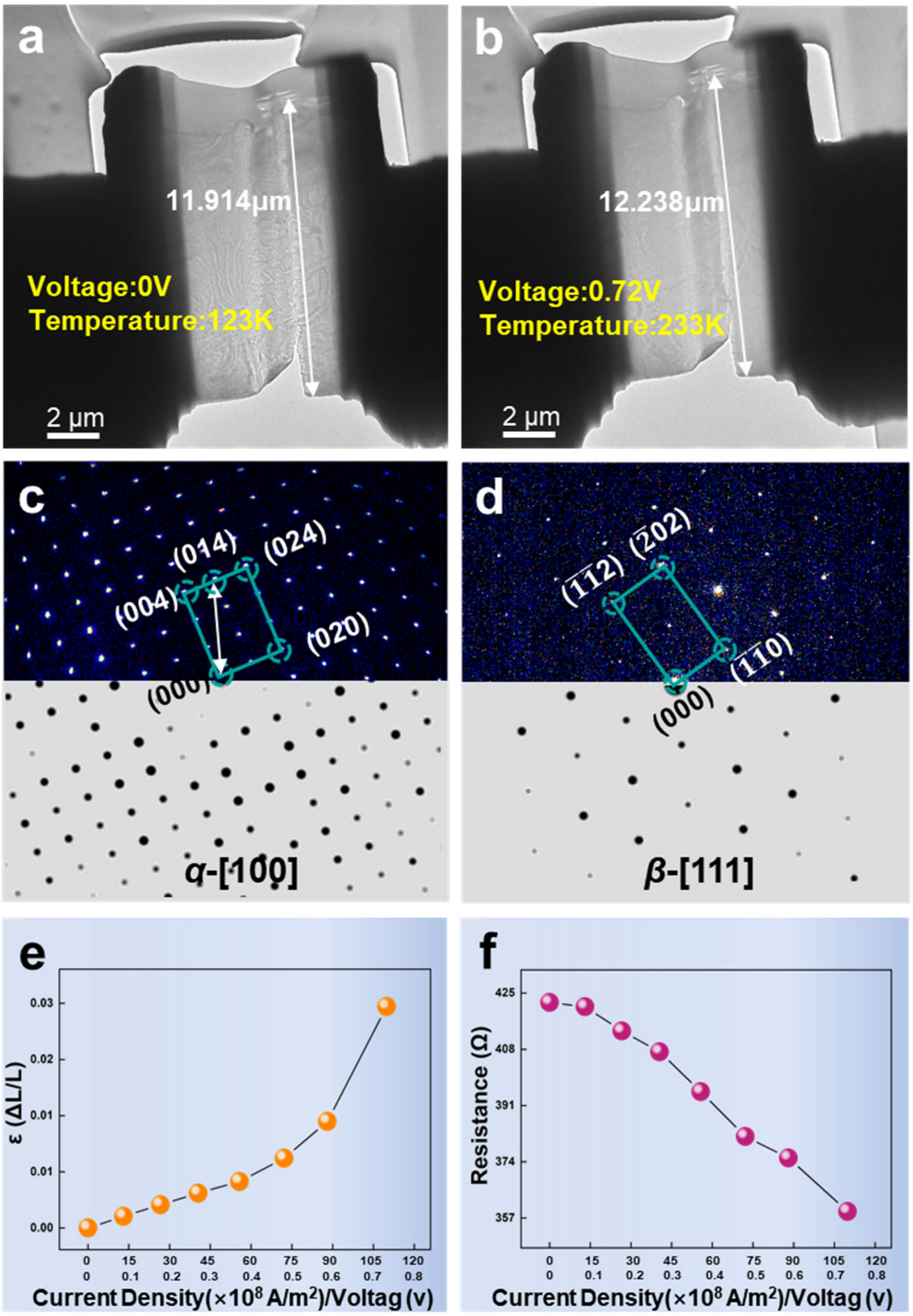

Figure S3. A giant electromechanical coupling effect is observed in Ag_2Se film at low temperature. (a) TEM image of the [100] oriented α-Ag_2Se at 0 V and 123 K (cooled by liquid N_2), where the length of the film along the [014] direction is measured. (b) TEM image of the same sample when the applied voltage is 0.72 V, where the measured temperature is 233 K and the length increases. (c), (d) The collected SAED patterns corresponding to (a) and (b), with comparison to the simulated ones, respectively. (e) The measured strain along the [014] direction versus the applied current density. (f) The measured resistance versus the applied current density.

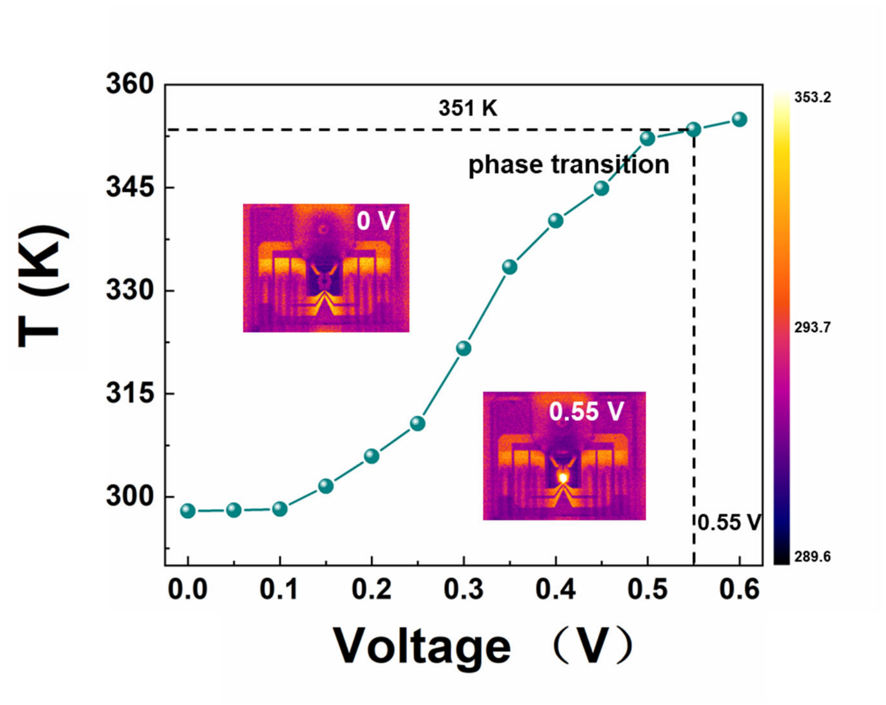

Figure S4. Measured temperature of Ag_2Se under the applied voltage due to Joule heating. The temperature of the Ag_2Se sample loaded on the chip is measured by high resolution infrared thermometer. It shows the temperature reaches only 351 K, when α-Ag_2Se has completely transformed into β-Ag_2Se, showing there exists athermal effect in the phase transition.

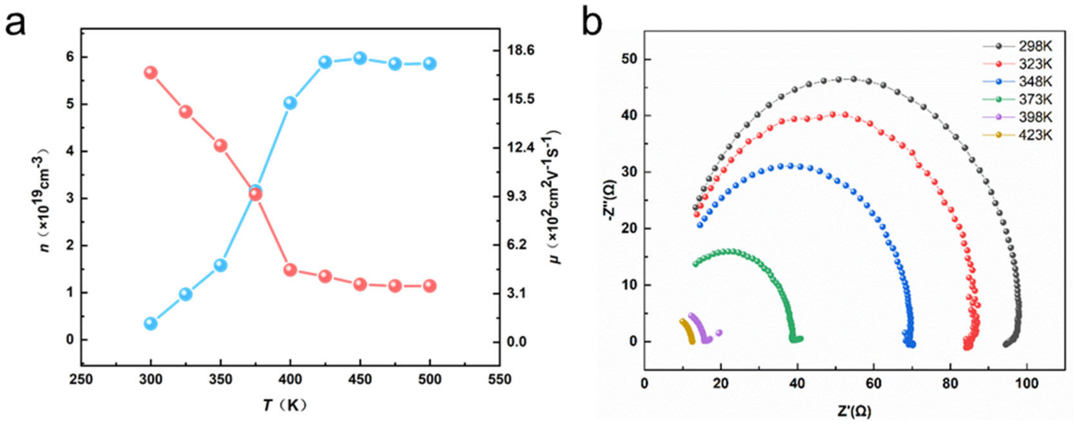

Figure S5. Carrier mobility/concentration and impedance diagram (a) The relationship between carrier concentration and carrier mobility with temperature. (b) Nyquist plot of Ag_2Se in the temperature range of 298K to 423K. Frequency range from 10^2Hz to 10^6Hz.

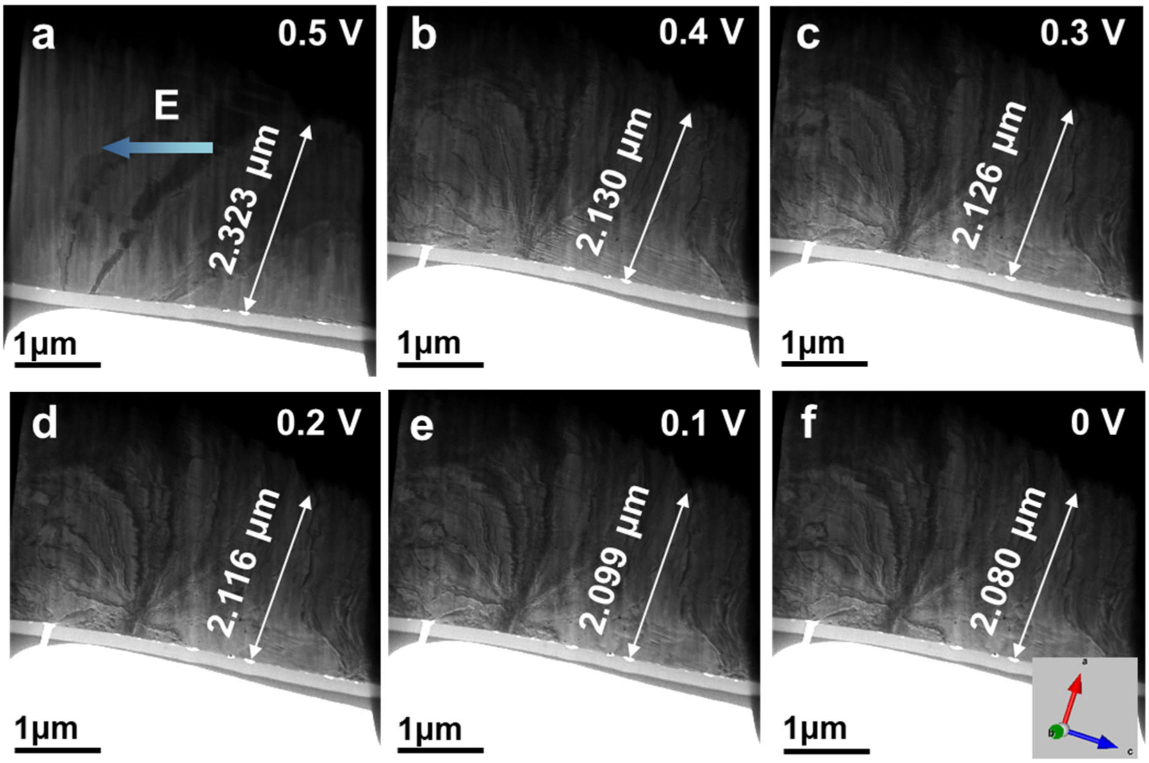

Figure S6. Microstructural evolution of Ag_2Se thin film under an decreasing electric field, showing a mechanic contraction along the [001] direction (referring to α-Ag_2Se). The TEM images taken when the applied voltage is (a) 0.5V, (b) 0.4 V, (c) 0.3V, (d) 0.2V, (e) 0.1V and (f) 0V, respectively. It shows ~11.8% strain at 0.5V, ~1.9% strain at 0.4V, ~1.7% strain at 0.3V, ~1.1% strain at 0.2V, and ~0.5% strain at 0.1V.

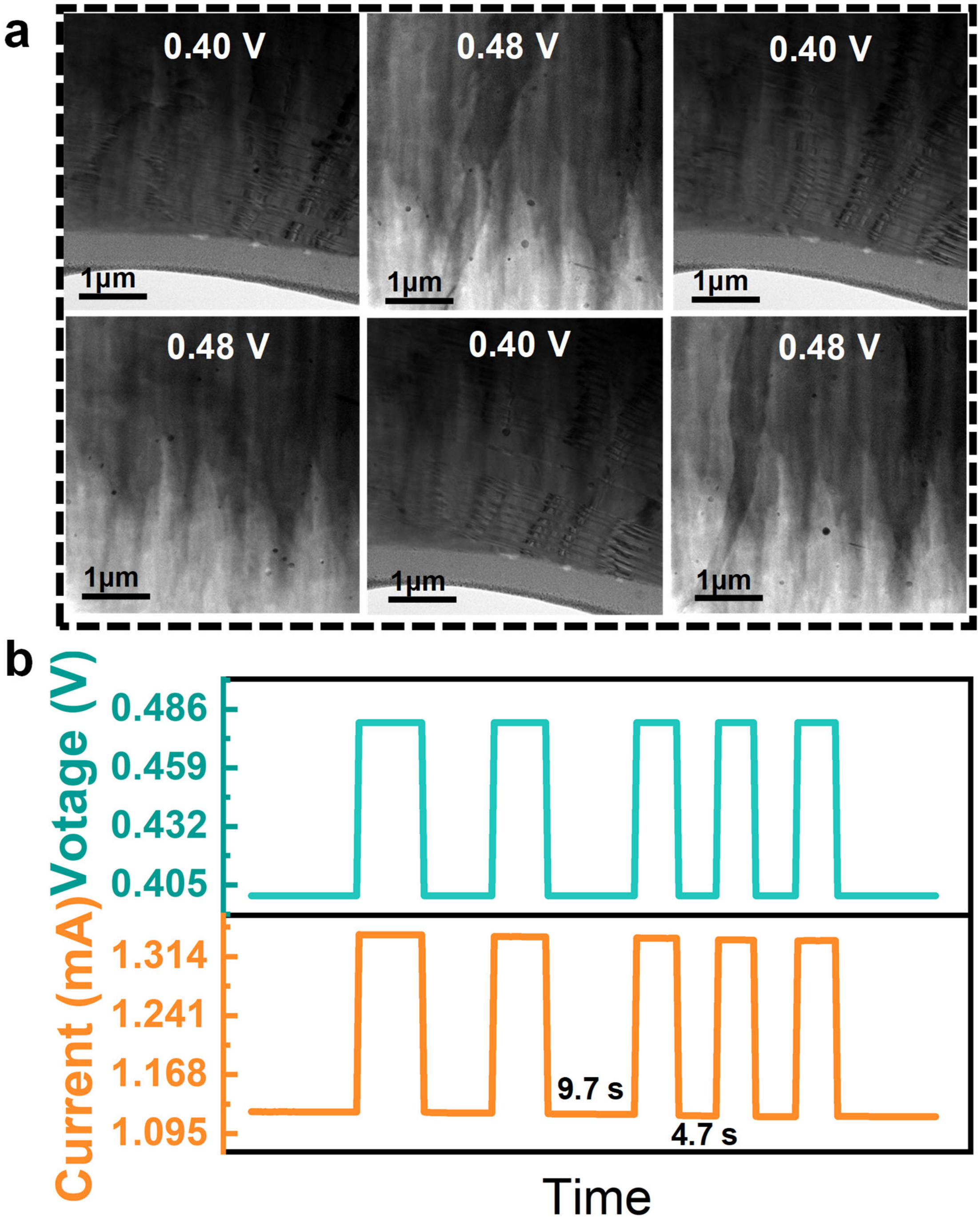

Figure S7. Switching in Ag_2Se between the expansion and contraction in response to electric pulses. (a) Stretch and contraction in the Ag_2Se film when the pulsed voltage is switched between 0.4 V and 0.48 V. (b) The pulsed voltage, as well as the corresponding loop current in the circuit, versus time. Such a swift and reversible mechanical displacement can be repeated many times.

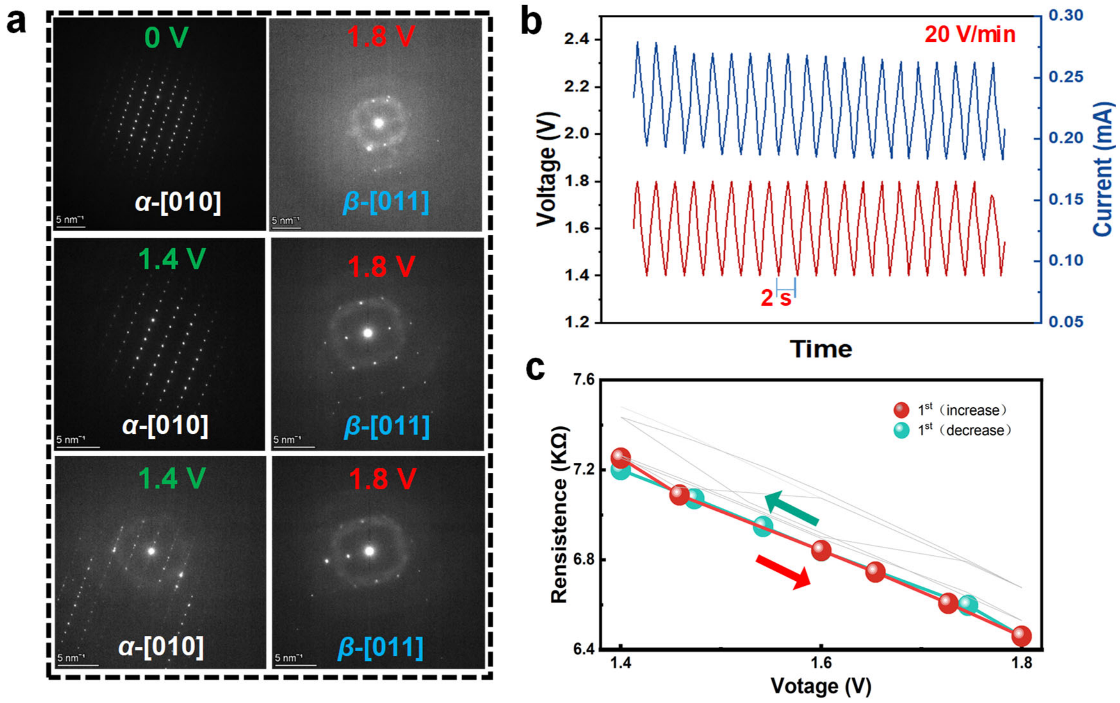

Figure S8. Switching in the Ag_2Se semiconductor between expansion and contraction in response to electric pulses. (a) Snapshots of the SAED patterns at different applied voltage, when a triangle-shape pulsed voltage (amplitudes between 1.4V and 1.8V) is applied, showing α-Ag_2Se at 1.4V, and β-Ag_2Se at 1.8V. (b) The pulsed voltage, as well as the corresponding loop current in the circuit, versus time. (c) Change of the resistance versus the applied triangle-shape voltage. Such a swift and reversible mechanical displacement can be repeated many times. The applied voltage in one experiment is different from the other, as the setup of the electrical chips and conductive path prepared by FIB during TEM sample preparation are different.

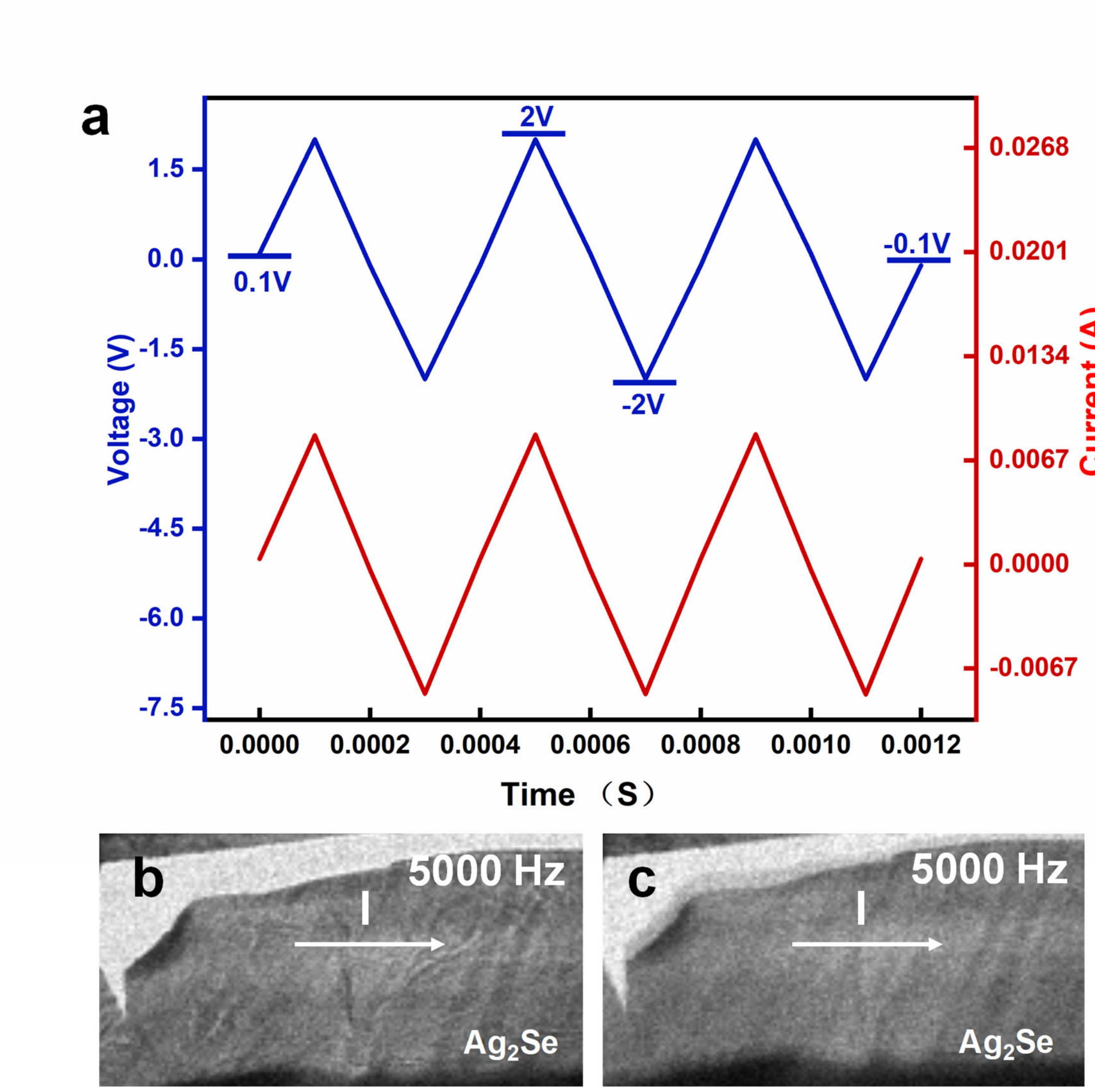

Figure S9. Fast switching of phase transition and resistance in Ag_2Se under the applied alternative current (AC) with frequency of 5000 Hz. (a) The pulsed voltage, as well as the corresponding loop current in the circuit, versus time. (b) The snapshots of the Ag_2Se thin crystal at 0V without application of AC current. (c) The snapshot of the Ag_2Se thin crystal under AC of 5000 Hz, while the image was recorded with the CCD with 50 ms exposure time, showing the shadow of the sample's elastic deformation.

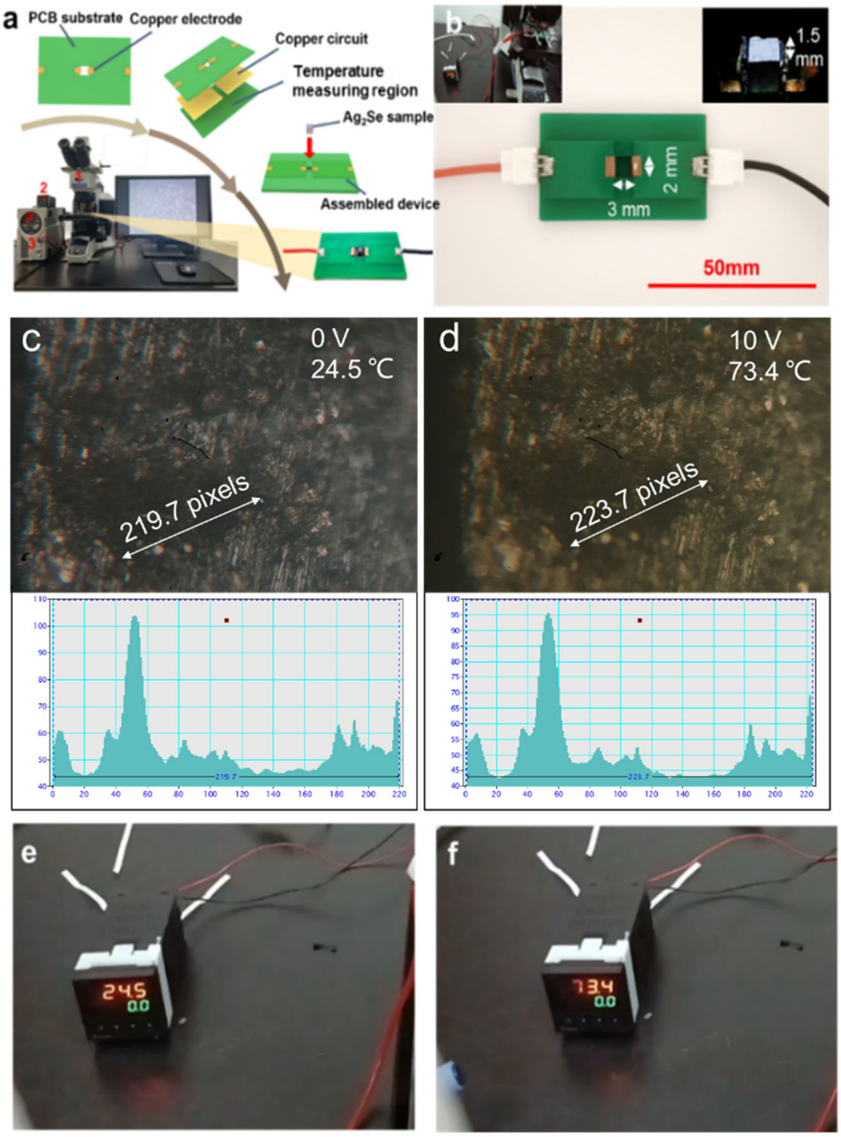

Figure S10. Macroscopic characterization of the elastic deformation of Ag_2Se under the applied voltage/current. (a) Experimental setup consisting of a copper clamp, a conductive copper (Cu) electrode in the middle and a thermocouple placed at the bottom. The deformation was captured using the optical microscope and a CCD camera. (b) The Ag_2Se block used in the testing has dimensions of 3 x 2 x 1.5 mm. (c) The morphology of the Ag_2Se sample before the application of the voltage. (d) The morphology of the Ag_2Se sample when the voltage of 10 V is applied， A significant strain was observed in the images. (e, f) Measured temperatures corresponding to figures (c) and (d).

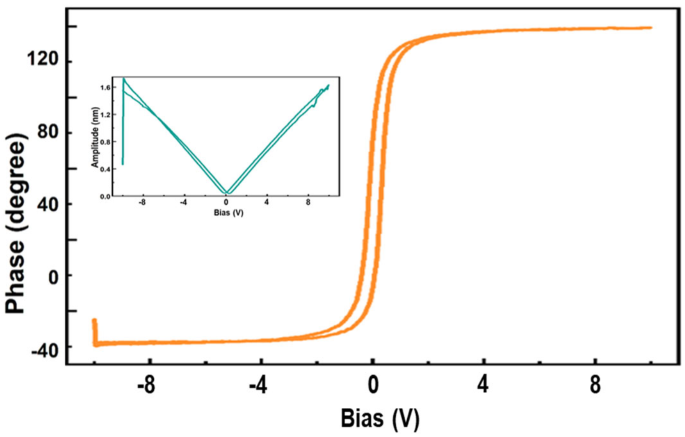

Figure S11. Amplitude and phase signal of PFM.

unit: Å	d_x	d_y	d_z	$\|d\|$
Se1	-0.019	-0.107	-0.412	0.426
Se2	0.019	0.107	-0.412	0.426
Se3	0.019	-0.107	0.412	0.426
Se4	-0.019	0.107	0.412	0.426

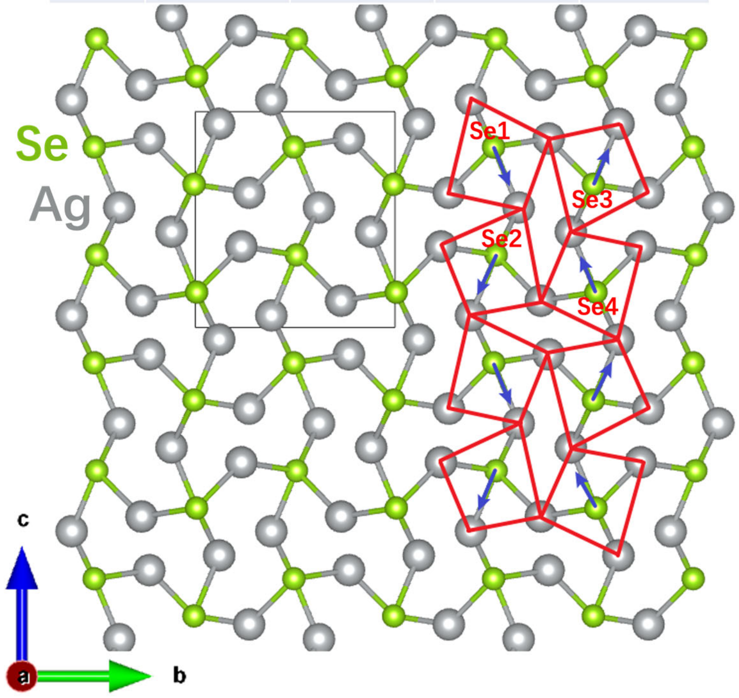

Figure S12. Ground-state structure of α-Ag_2Se optimized with first-principles density functional theory. The table reports the value of the local displacement (blue arrow) of the Se anion relative to the center of the surrounding Ag_4 tetrahedron (red lines) in the ground-state structure of α-Ag_2Se.

Computational Methods

We perform density functional theory calculations with QUANTUM ESPRESSO (QE) [1] using PBEsol exchange-correlation functional [2]. Ultrasoft pseudopotentials taken from the Garrity-Bennett-Rabe-Vanderbilt (GBRV) library [3] are used for structural optimizations. We use an energy convergence threshold of 10^{-7} Ry, a force convergence threshold of 10^{-6} Ry/bohr, and a plane wave cutoff energy of 60 Ry to obtain the ground-state structure. An $8 \times 8 \times 8$ k-point mesh is used for Brillouin zone integration.

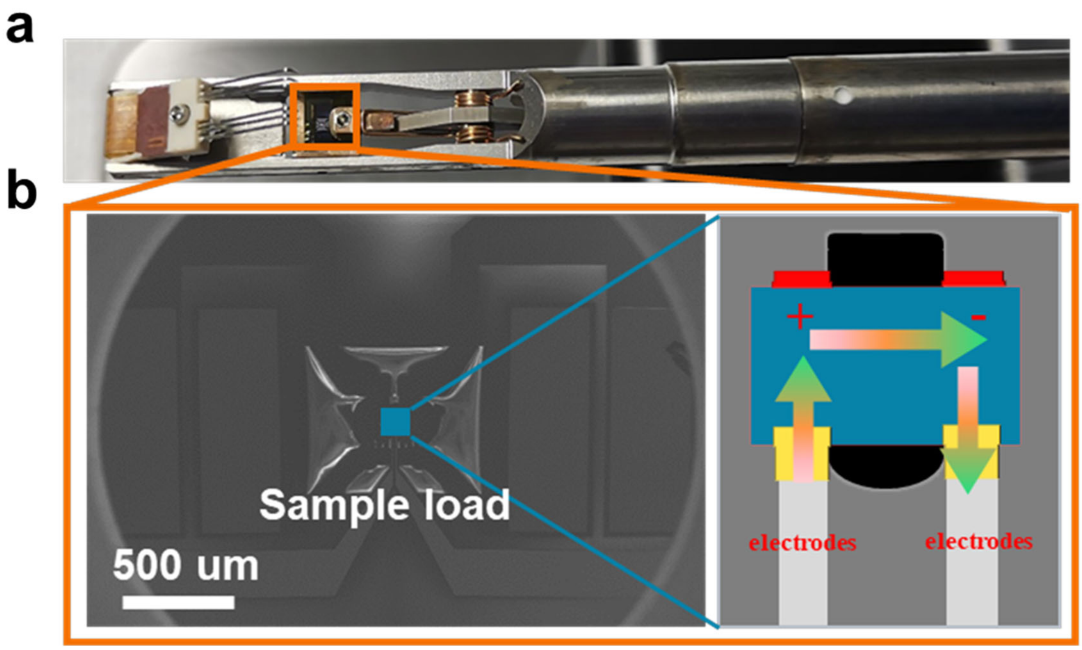

Figure S13. Experimental setup for the *in-situ* STEM and TEM with samples mounted on the biasing chip. (a) A picture of the front part of the electric biasing holder used in TEMs. (b) An enlarged view of the tip where a thin TEM sample prepared by FIB can be loaded and an illustration showing a bias can be applied and the current can be measured while the microstructural evolutions is imaged by TEM.

unit: Å	$\boldsymbol{d_x}$	$\boldsymbol{d_y}$	$\boldsymbol{d_z}$	$\lvert\boldsymbol{d}\rvert$
Se1	0.022	0.107	-0.403	0.417
Se2	-0.019	-0.105	-0.405	0.419
Se3	-0.016	0.109	0.422	0.436
Se4	0.018	-0.106	0.421	0.434

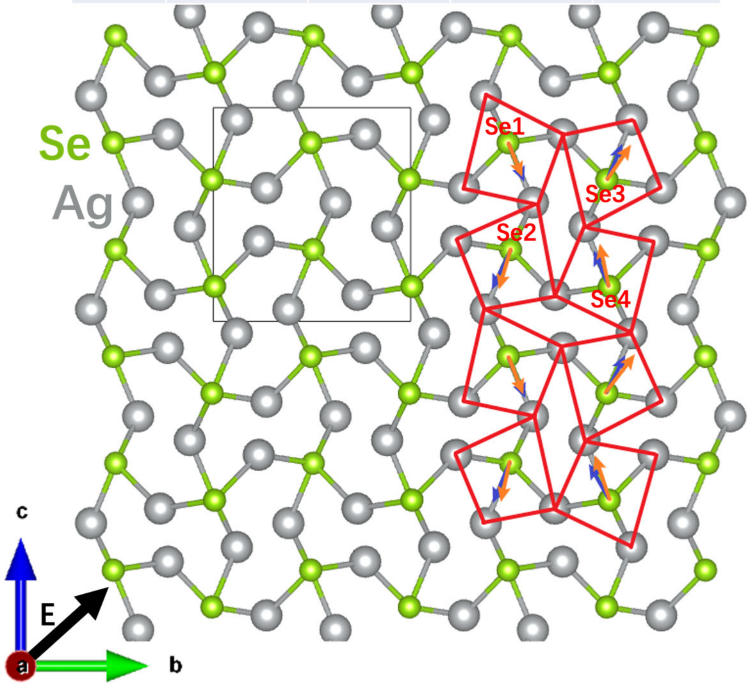

Figure S14. Structure of α-Ag_2Se under an applied external electric field optimized with first-principles density functional theory. The table reports the value of the local displacement (orange arrow) of the Se anion in the presence of an electric field. The blue arrows represent zero-field local displacements.

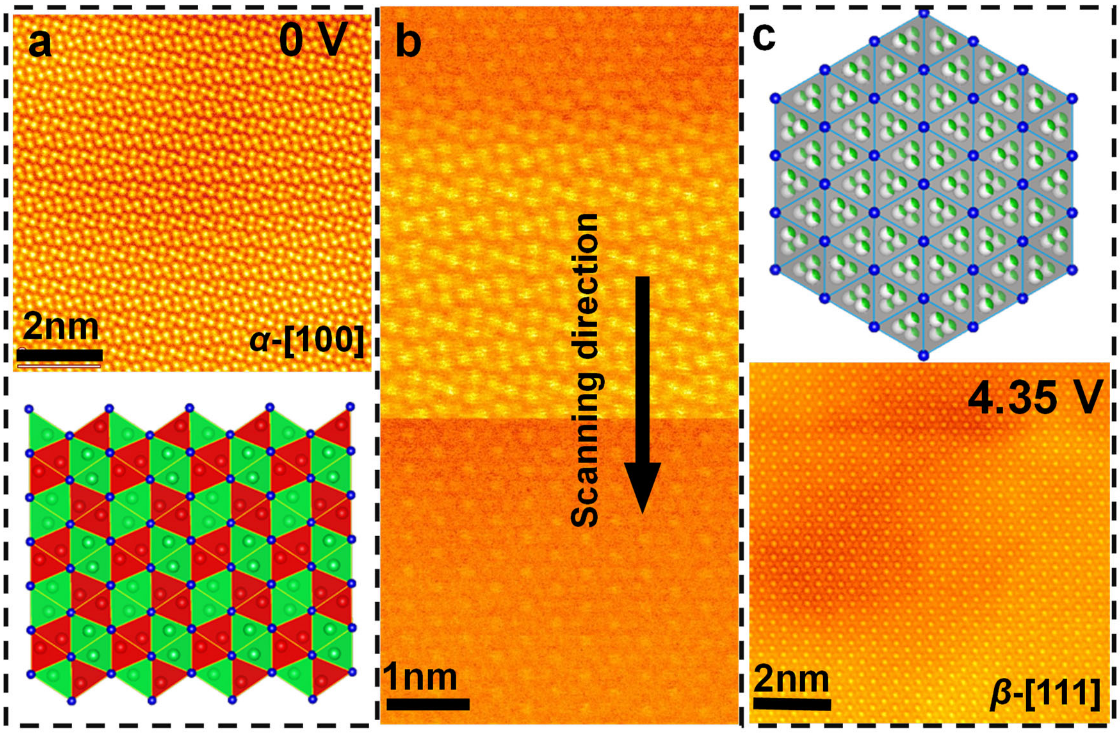

Figure S15. An electrically induced phase transition from the low-symmetry *α*-Ag_2Se to the high-symmetry *β*-Ag_2Se studied by *in-situ* STEM. (a) A HAADF-STEM image of *α*-Ag_2Se along the [100] zone axis and an illustration of its atomic structural model. (b) A HAADF-STEM images collected during the phase transition from *α*-Ag_2Se to *β*-Ag_2Se. (c) A HAADF-STEM image of *β*-Ag_2Se along the [111] zone axis and an illustration of its atomic structural model.

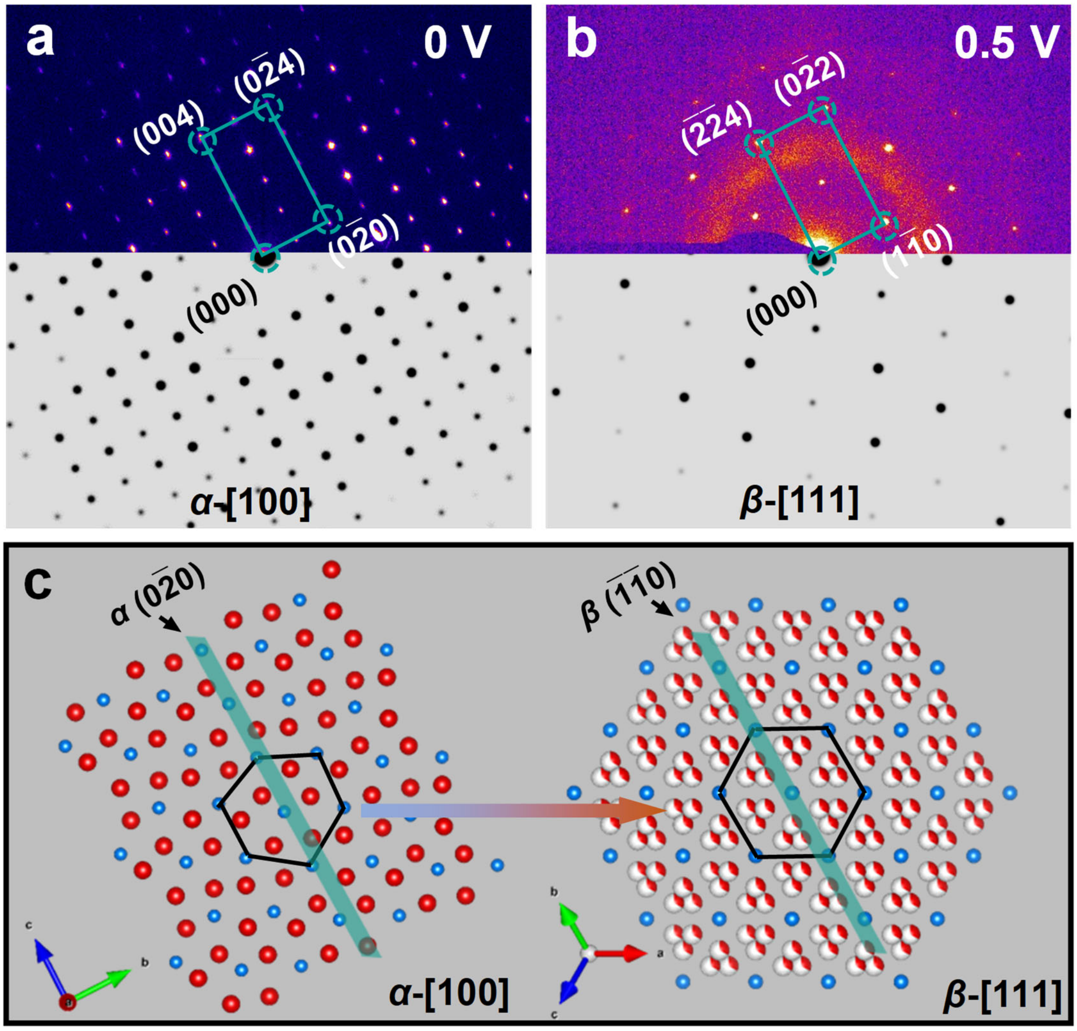

Figure S16. An electrically induced phase transition from low-symmetry *α*-Ag_2Se to high-symmetry *β*-Ag_2Se studied by *in-situ* SAED. (a) A SAED pattern collected at 0V (up), which can be indexed as *α*-Ag_2Se along the [100] zone axis, compared to the simulated SAED pattern (down). (b) A SAED pattern collected at 0.5V (up), which can be indexed as *β*-Ag_2Se along the [111] zone axis, compared to the simulated SAED pattern (down). (c) An illustration of their atomic structural model during the phase transition.

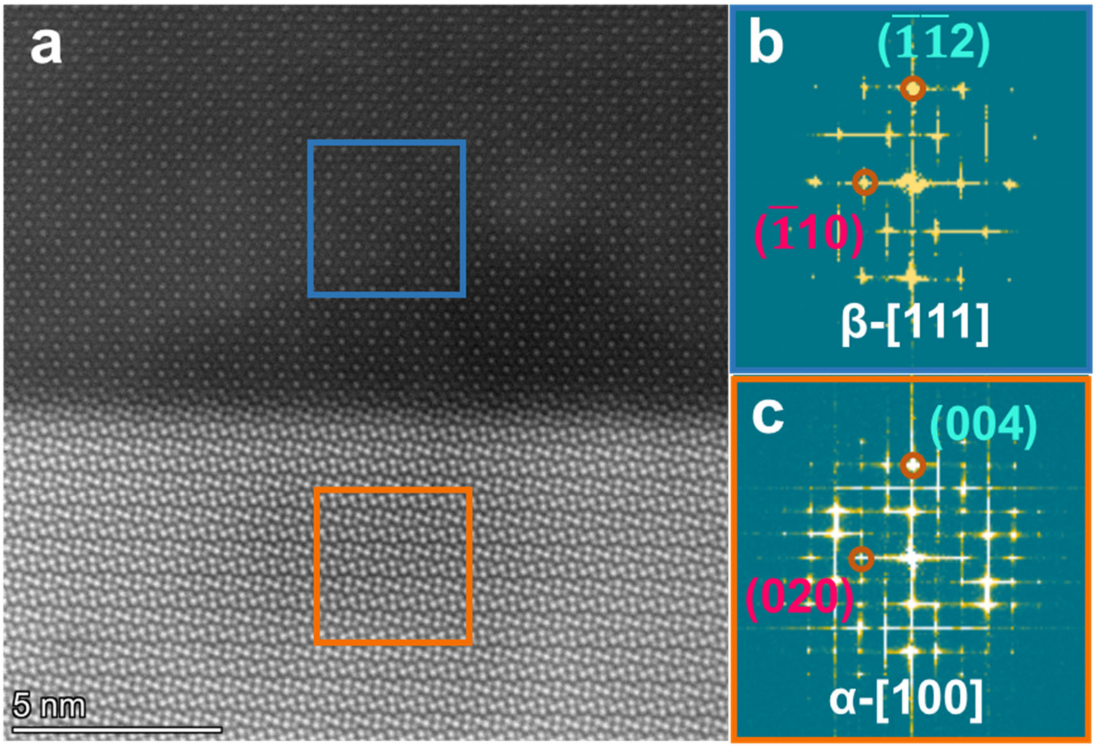

Figure S17. Structural characterization of the interface between the [100] *α*-Ag_2Se and the [111] *β*-Ag_2Se in the electrically induced phase transition. (a) An atomic resolution HAADF-STEM image of the two-phase interface collected when the applied voltage is 0.5V. (b) The FFT pattern of the red area of (a), which can be indexed as *β*-Ag_2Se along the [111] zone axis. (c) The FFT pattern of the orange area of (a), which can be indexed as *α*-Ag_2Se along the [100] zone axis.

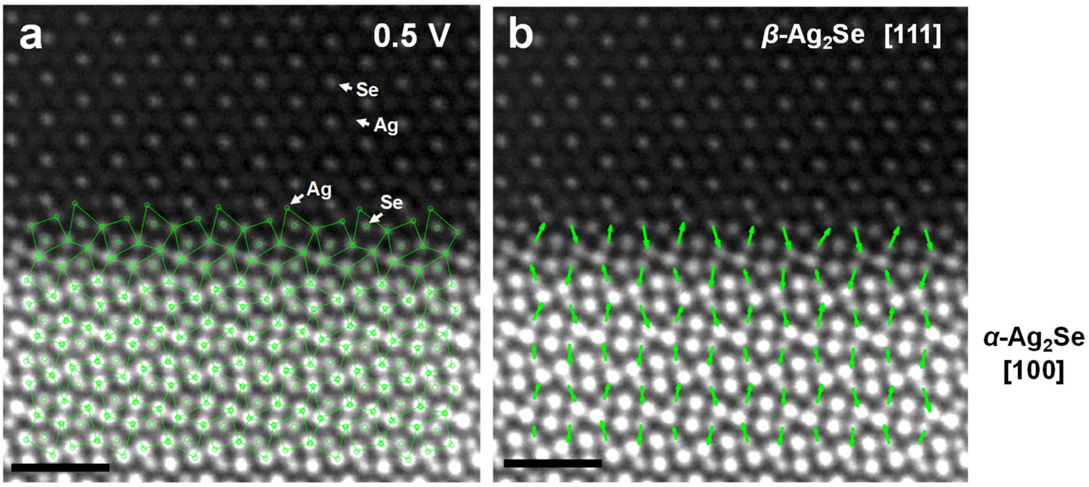

Figure S18. Identifying the polarizations in α-Ag_2Se during α-to-β phase transition when the applied voltage is 0.5V. (a) A HAADF STEM image of the two-phase interface during the phase transition, in which the Ag^+-tetrahedron is outlined in α-Ag_2Se so that the polarizations can be experimentally measured. (b) The HAADF STEM image overlapped with the vectors of the polarizations, showing an increment of the polarizations at the interface. The scale bar is 1 nm.

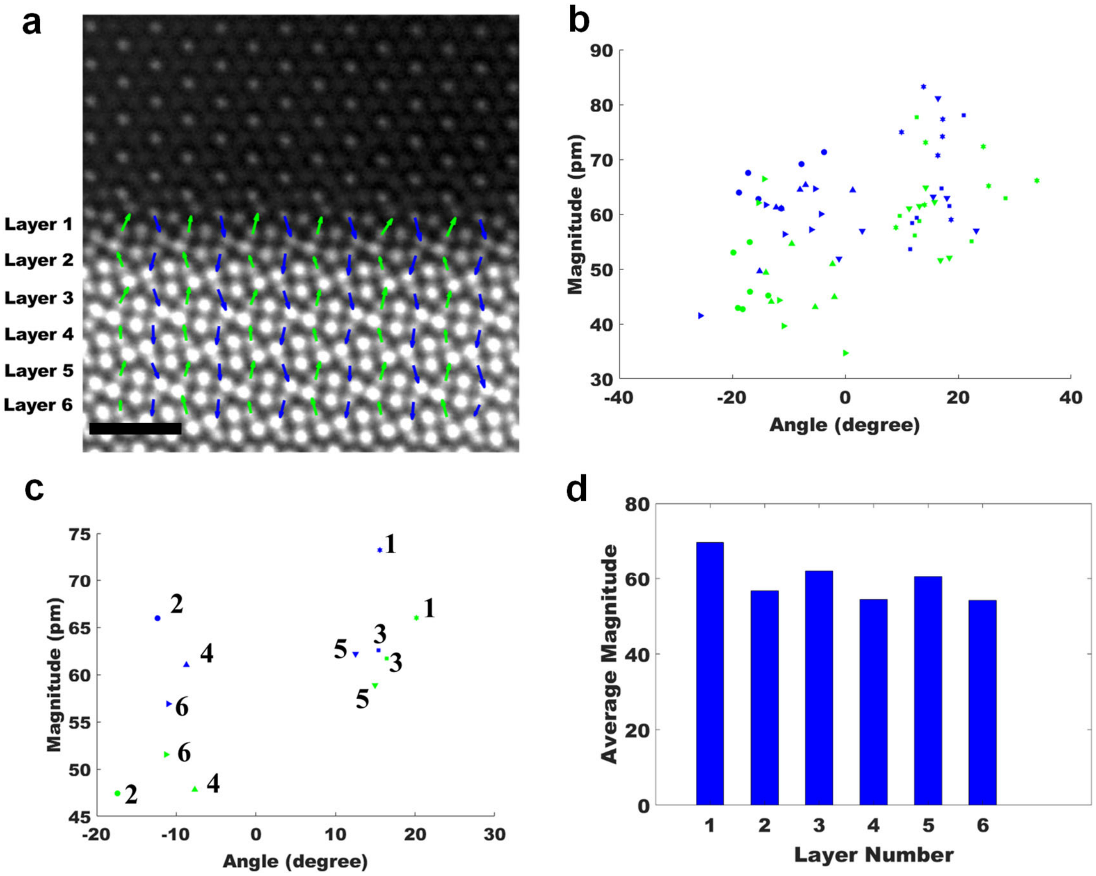

Figure S19. The statistical analysis of the polarizations in α-Ag_2Se during α-to-β phase transition when the applied voltage is 0.5V. (a) The HAADF STEM image overlapped with the vectors of the polarizations of α-Ag_2Se, in which α-Ag_2Se is separated as 6 layers and the polarizations are categorized as those pointed up or down. (b) A statistical analysis showing the distribution of the magnitude and angle of the polarizations. (c) A statistical analysis of the average magnitude and angle of the polarizations in 1st layer to the 6th layer. (d) A histogram of the average magnitude of the polarizations from 1st layer to the 6th layer, showing an enlargement of the magnitude in the interface (1st layer). The scale bar is 1 nm.

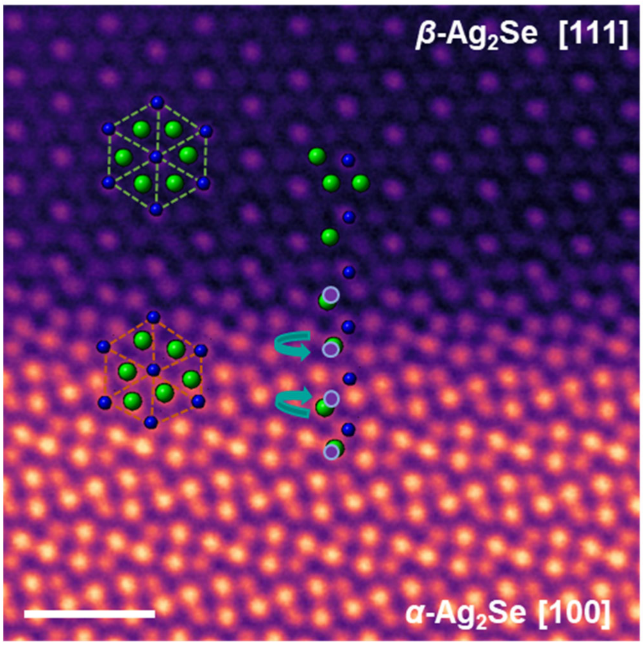

Figure S20. Direct observation of Ag^{+} ions diffusing into neighboring Ag^{+} vacancies under a high electric field. This is an atomic resolution HAADF-STEM image of the interface between the [100] α-Ag_2Se and the [111] β-Ag_2Se collected when the applied voltage is 0.5V. The Ag^{+}-ions are represented as green circles, Se^{2-}-ions as blue circles, and Ag^{+} vacancies by crayon circles. The hopping of the Ag^{+} ions into neighboring Ag^{+} vacancies can be seen. Such a diffusion lead to the collapse of the spontaneous polarizations in α-Ag_2Se and triggers the phase transition to β-Ag_2Se.

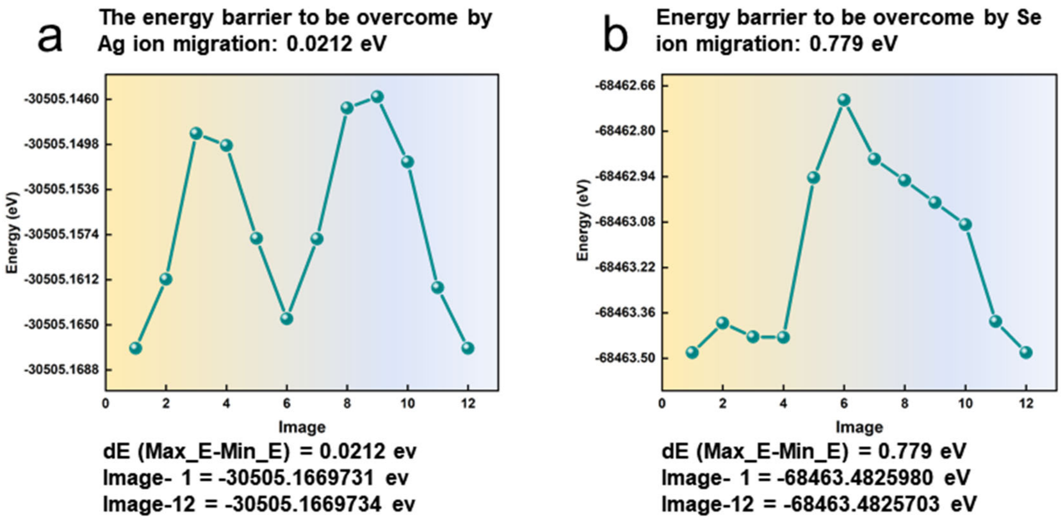

Figure S21. DFT calculations show that migration barrier of Ag-ion is ~37 times smaller than that of Se. (a) The energy barrier of Ag-ion's migration. (b) The energy barrier of Se-ion's migration.

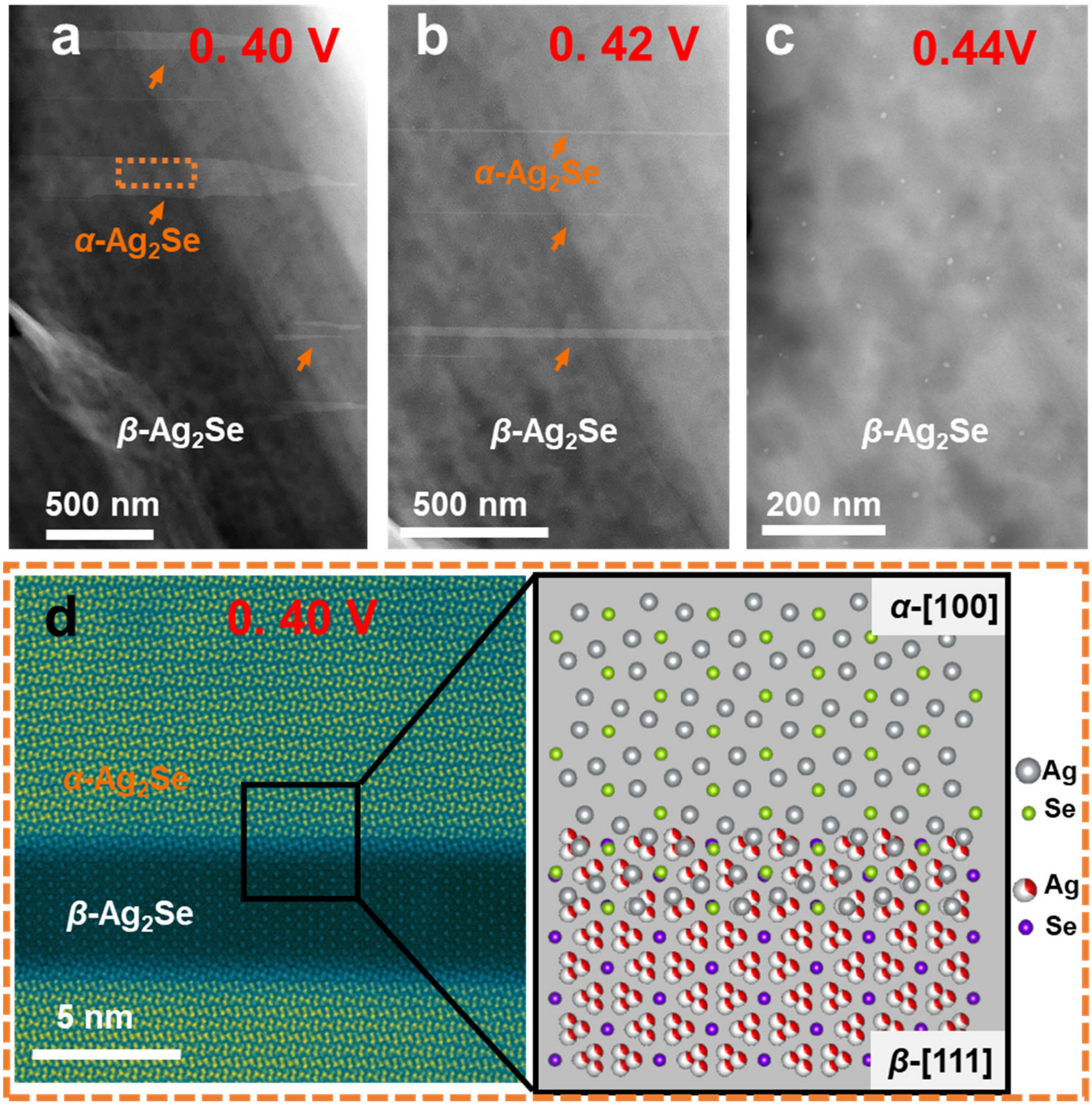

Figure S22. Nano-sized heterogeneous phase slabs formed during the electrically induced phase transition. (a) HAADF STEM image of slabs of α-Ag_2Se embedded in the β-phase matrix, when the majority of the sample has transformed into the β-phase under 0.4V. (b) The slabs of α-Ag_2Se embedded in the β-phase matrix become thin, when the applied voltage is increased to 0.42V. (c) The whole sample has completely transformed into the β-phase, when the voltage reaches 0.435V. (d) An enlarged view of the orange rectangle area in (a) and an illustration of the corresponding atomic structure model of the interface, showing that inside the α-Ag_2Se slab, a thin layer of β-phase can still be found, indicating the complex and heterogenous structure formed during the phase transformation.

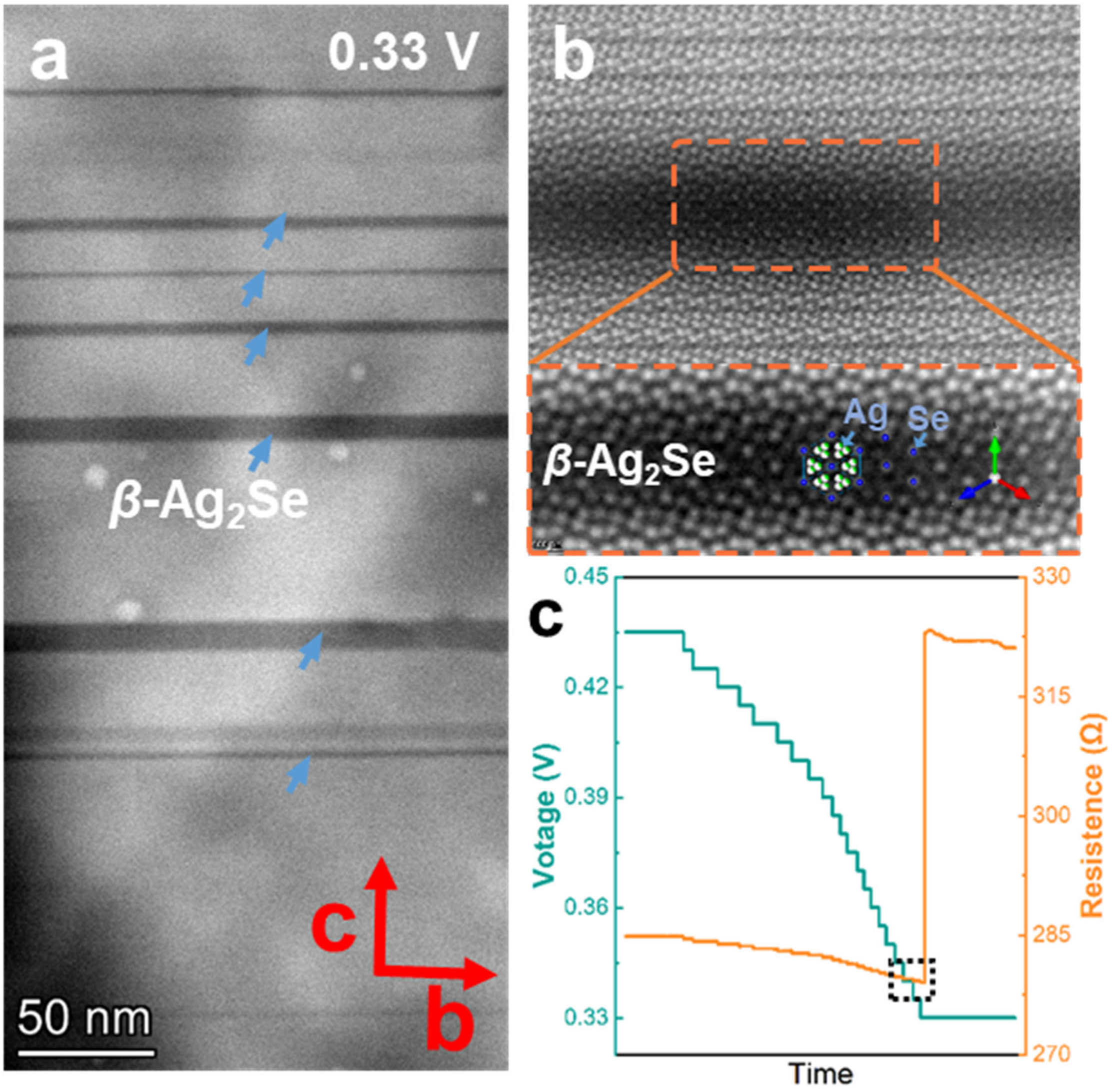

Figure S23. Nano-sized heterogeneous phase slabs formed during the β-to-α phase transition when the applied voltage is decreased. (a) When the voltage is decreased to 0.33V, the HAADF STEM image of slabs of β-Ag_2Se embedded in the α-phase matrix, when the majority of the sample has transformed from β to α phase. (b) The high magnification HAADF STEM image of the slab of β-Ag_2Se embedded in the α-phase matrix at 0.33 V. (c) Change of the resistance and the decreased voltage, showing there is still an abrupt increase in the resistance when the phase transition occurs in the majority of sample, despite the existence of the β slabs.

Supplementary Note 1:

There is a difference between "resolution" and "precision". The first one is determined by the instrument and gives the minimal distance that two atoms can be seen as separate, the latter is the precision in measurement that one can get for the shift of two atoms with respect to each other. We determined the length of the dipole moment involves calculation of the distance between the virtual center of the Ag^{+}-ions and Se^{2-}. This is done through a fitting process using Gaussian function to find the atomic centers. The calculated lengths are then averaged to calculate the polarization (with the variation provided). By employing this method, it is possible to get a polarization value much smaller than the resolution limit of a STEM.

Supplementary References:

[1] J. Phys.: Condens. Matter 21, 395502 (2009).

[2] Phys. Rev. Lett. 100, 136406 (2008).

[3] Comput. Mater. Sci. 81, 446 (2014).
