# Supplementary material for: Current induced electromechanical strain in thin antipolar Ag2Se semiconductor
Source: Nat Commun. 2025 Feb 20;16:1818. doi: 10.1038/s41467-025-57057-5 (PMC11842724; doi:10.1038/s41467-025-57057-5)
Supplement: Supplementary file 2 — Description Of Additional Supplementary File [file 41467_2025_57057_MOESM2_ESM.pdf]

Description of Additional Supplementary Files：

Supplementary Movies 1. *In-situ* TEM of the electrically induced mechanic stretch and corresponding microstructural evolution of Ag_2Se thin film under an increasing electric field. The applied voltage was slowly increased from 0 V to 0.5 V (×5 times faster than the real speed).

Supplementary Movies 2. *In-situ* TEM of the electrically induced mechanic stretch of Ag_2Se thin film under an increasing electric field at low temperature cooled by liquid N_2. The applied voltage was slowly increased from 0 V to 0.72 V (×1 times faster than the real speed).

Supplementary Movies 3. *In-situ* TEM of the electrically induced mechanic contraction and corresponding microstructural evolution of Ag_2Se thin film under a decreasing electric field. The applied voltage was slowly increased from 0.5V to 0V (×5 times faster than the real speed).

Supplementary Movies 4. *In-situ* TEM of the electrically induced mechanic stretch and corresponding microstructural evolution of Ag_2Se thin film under a negative bias. The applied voltage was slowly changed from 0V to -0.5 V (×5 times faster than the real speed).

Supplementary Movies 5. *In-situ* TEM of the fast switching of Ag_2Se between expansion and contraction in response to rectangular shape electric pulses. The pulsed voltage is switched between 0.4 V and 0.48 V (×7.5 times faster than the real speed).

Supplementary Movies 6. *In-situ* TEM of the fast switching of Ag_2Se between expansion and contraction in response to triangle-shape electric pulses. The pulsed voltage is switched between 1.4 V and 1.8 V (×7.5 times faster than the real speed).

Supplementary Movies 7. *In-situ* SAED showing the fast phase transition in Ag_2Se thin film in response to triangle-shape electric pulses. α-Ag_2Se is identified at 1.4V, and at 1.8V β-Ag_2Se forms. The pulsed voltage is switched between 1.4 V and 1.8 V (×7.5 times faster than the real speed).

Supplementary Movies 8. *In-situ* TEM showing the fast electroelastic deformation of Ag_2Se thin film in response to alternative electric current with frequency of 20 Hz. α-Ag_2Se is identified at 0V, and at 2V β-Ag_2Se forms. The pulsed voltage is switched between 0 V and 2V (×1 times faster than the real speed).

Supplementary Movies 9. *In-situ* STEM of the electrically induced α-to-β phase transition observed at atomic resolution. The applied voltage was slowly increased from 0.42V to 0.435 V (×2 times faster than the real speed), in which an instant phase transition can be observed.
